# Development and psychometric testing of symptom severity scale in older patients with cardiometabolic multimorbidity

**DOI:** 10.1186/s12877-025-06370-1

**Published:** 2025-10-14

**Authors:** Merve Gulbahar Eren, Havva Sert

**Affiliations:** Deparment of Internal Medicine Nursing, Faculty of Health Science, Sakarya University, Sakarya, Turkey

**Keywords:** Validity and reliability, Cardiometabolic multimorbidity, Scale, Symptoms, Older patients

## Abstract

**Background:**

Given the high prevalence of multiple cardiometabolic disorders in the older population and their negative impact on quality of life, assessing symptom burden is of critical importance. No assessment tool is available to holistically measure the severity of common symptoms in cardiometabolic diseases. This study aimed to develop the “Symptom Severity Scale in Patients with Cardiometabolic Multimorbidity (SSS-CM)” and to perform psychometric testing in the Turkish older population.

**Methods:**

This methodological study was conducted between August and November 2024 with patients (*n* = 388) aged ≥ 65 years with at least two cardiometabolic diseases (coronary heart disease, hypertension, diabetes mellitus, stroke, and dyslipidemia) who were followed up in the internal medicine, cardiology, and neurology clinics of a training and research hospital. Data was collected using the Patient Information Form, the SSS-CM, and the EQ-5D-3 L scale. The validity and reliability of the scale were tested using exploratory and confirmatory factor analyses, Cronbach’s alpha analysis, and Pearson correlation analysis.

**Results:**

The exploratory factor analysis determined that the scale had a single-factor structure explaining 65.753% of the total variance, and the factor loads ranged between 0.658 and 0.898. In confirmatory factor analysis, χ2/df = 1.739. Cronbach’s alpha internal consistency coefficient was found to be 0.978. Pearson correlation analysis showed a significant negative correlation between symptom severity and overall quality of life.

**Conclusions:**

The SSS-CM is a valid and reliable measurement tool for assessing symptom severity in cardiometabolic older patients with complex care and treatment needs. This scale could contribute to assessing the effectiveness of symptom management interventions to alleviate symptoms and improve the quality of life among the older population. However, the findings should be interpreted with caution due to the purposive sampling from a single center setting in Turkey, which may limit the generalizability across different populations and healthcare systems.

**Clinical trial number:**

Not applicable.

**Supplementary Information:**

The online version contains supplementary material available at 10.1186/s12877-025-06370-1.

## Introduction

Multimorbidity is becoming a global health problem associated with reduced quality of life and increased utilization of healthcare resources [1]. Cardiometabolic multimorbidity, one of the main profiles of multimorbidity, is the coexistence of two or more cardiometabolic diseases, including diabetes, hypertension, ischemic heart disease, and stroke [1–3]. Cardiometabolic multimorbidity is becoming increasingly common as the population ages, with 4.7% of adults aged 60 years and older having at least two comorbid cardiometabolic diseases [3, 4].

As the severity of symptoms increases in patients with cardiometabolic multimorbidity, the morbidity and mortality rates increase, and the prognosis worsens [3, 5, 6]. Multiple factors influence the development of physical symptoms in patients with cardiometabolic multimorbidity [5, 6]. Common risk factors and similar pathogenic mechanisms are interconnected and shape this process [3, 5]. Chest pain, dyspnea, heart palpitations, fatigue, weakness, dizziness, syncope, anxiety, restlessness, and sleep problems are common symptoms in patients with cardiovascular diseases that affect their daily activities and quality of life [1, 7, 8]. The presence of comorbid conditions such as diabetes, hypertension, and stroke may increase the severity of these symptoms [7, 8]. It may additionally lead to symptoms such as confusion, memory problems, blurred/double vision, balance and coordination problems, loss of strength/sensation in the arms or legs, and numbness/tingling in the hands or feet.

Symptoms common to cardiometabolic multimorbidity diseases lead to a decrease in functional capacity, difficulty in performing activities of daily living, increased care dependency, repeated hospitalizations, and, because of all these effects, a decrease in quality of life [1, 9, 10]. Klompstra et al. [11] reported that higher symptom burden was associated with lower health-related quality of life. An adverse change in symptom burden (experiencing higher symptom burden) over a two-year follow-up period decreased health-related quality of life for one year [11]. Considering all these effects, assessing the presence and severity of the most common symptoms specific to cardiometabolic diseases and the effects of each symptom on patients becomes an important requirement in providing comprehensive care to patients with cardiometabolic multimorbidity, reducing readmissions and improving their quality of life.

Symptom burden is a critical determinant of health outcomes in older adults with cardiometabolic multimorbidity [8]. Regarding literature, despite the existence of several symptom assessment tools, such as the Edmonton Symptom Assessment Scale (ESAS) [12], the Memorial Symptom Assessment Scale (MSAS) [13], and the MD Anderson Symptom Inventory (MDASI) [14], these instruments were primarily developed for use in oncology or palliative care populations. Accordingly, these symptom assessment scales primarily focus on general symptoms such as pain, dry mouth, and low energy, rather than measuring symptoms specific to patients with cardiometabolic multimorbidity. Consequently, they may not adequately capture the unique symptom profiles of individuals with coexisting cardiovascular, cerebrovascular, and metabolic conditions.

On the other Hand, different scales are specifically designed for each disease included in cardiometabolic multimorbidity. Examples of symptom assessment scales include the Type 2 Diabetes Symptom Checklist [15], the Cardiac Symptom Survey for coronary artery bypass grafting [16], the Ischemic Stroke Symptoms Questionnaire [17] and the Cardiovascular Symptoms Scale [18]. Since assessing patients with cardiometabolic multimorbidity requires using all these scales, it may lead to an increased workload and loss of time. Additionally, the geriatric population may have difficulty answering these extensive questionnaires separately. As a result, many of the available tools are lengthy or include domains not directly applicable to multimorbidity care, which may reduce feasibility in busy clinical environments and among frail or cognitively impaired patients.

In conclusion, it is noteworthy that no assessment tool is available to measure the severity of common symptoms in cardiometabolic diseases specifically adapted for older adults in the Turkish population. In addition, the measurement tools currently available in the literature have been limited in evaluating the common symptoms in cardiometabolic multimorbidity patients and their health outcomes. Therefore, symptom assessment of patients with cardiometabolic diseases is not performed holistically. This study aimed to develop and test the psychometric validity of the “Symptom Severity Scale in Patients with Cardiometabolic Multimorbidity (SSS-CM),” a comprehensive assessment tool designed for health professionals to easily use in evaluating the symptoms of older patients with cardiometabolic multimorbidity. The developed scale is expected to contribute significantly to the literature by assessing common symptoms in cardiometabolic multimorbidity, tracking changes in these symptoms over time, and evaluating their impact on patient outcomes from a subjective perspective. Additionally, the scale is anticipated to benefit symptom assessment for advanced clinical studies focusing on this population with complex care and treatment needs.

## Methods

### Design and participants

**Fig. 1:**
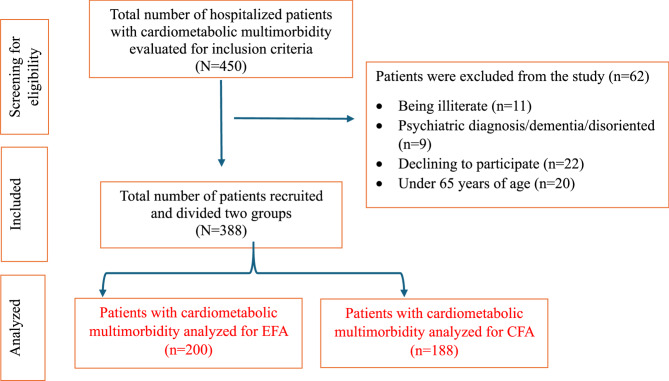
Flow diagram of the study sample

This study used a methodological design to evaluate the validity and reliability of the SSS-CM. This study was conducted between August and November 2024 with patients hospitalized in the Internal Medicine, Cardiology, and Neurology clinics of a training and research hospital in Türkiye. The study sample included patients (1) over the age of 65, (2) diagnosed with at least two cardiometabolic diseases (coronary heart disease, hypertension, diabetes mellitus, stroke, and dyslipidemia), (3) being conscious and able to communicate verbally, and (4) who volunteered to participate in the study. The purposive sampling method was used in this study. Regarding literature, the sample size recommended for scale development varies depending on the items, number of factors, and method [19]. A commonly recommended approach is to select at least 5–10 participants for each item [19]. The minimum recommended sample size for exploratory factor analysis (EFA) is 100 participants, and ideally 150–400 participants [20]. The sample size required for confirmatory factor analysis (CFA) should be 100–200 participants [21]. In this context, this study was conducted with 388 patient samples. After data collection was completed, the total sample (*N* = 388) was randomly divided into two independent subsamples for factor analysis. This random allocation was conducted during the analysis phase using SPSS’s random number generator function. Subsample 1 (*n* = 200) was used for EFA, and Subsample 2 (*n* = 188) was used for CFA (Fig. 1).

In psychometric research, it’s a widely endorsed practice to divide a dataset into two independent subsamples when conducting both EFA and CFA [22, 23]. This strategy enhances the validity of the factor structure by mitigating overfitting and ensuring that the model identified through EFA is not merely a product of sample-specific characteristics. Lorenzo-Seva et al. [22] emphasize that using the same sample for both EFA and CFA is undesirable, as it may lead to biased conclusions. They advocate for splitting the sample to obtain two equivalent subsamples, ensuring that each analysis is conducted on independent data [22]. Accordingly, the use of independent subsamples enhances the validity and generalizability of the resulting factor structure [22, 24].

### Stages of development of the SSS-CM

**Fig. 2:**
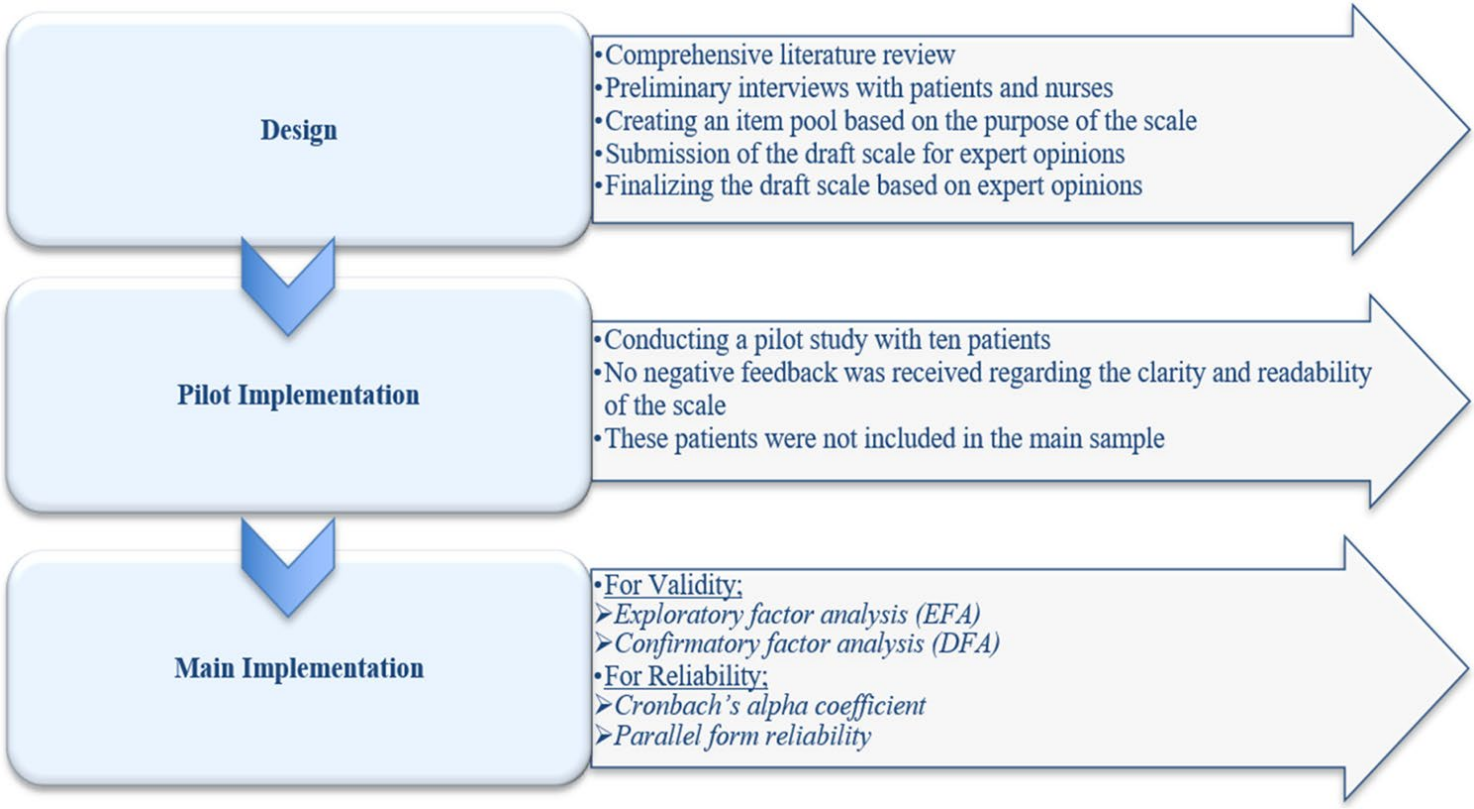
Stages of scale development

The development of the scale was carried out in three steps: design, pilot implementation, and main implementation stages (Fig. 2).

#### Design stage

The researchers prepared a pool of items for common symptoms of cardiometabolic multimorbidity through a literature review and preliminary interviews with five nurses caring for cardiometabolic patients and five patients. The authors of this study were nurse academicians with clinical and research experience in cardiovascular diseases. Additionally, the authors have received training in scale development and have published articles internationally in this field.

In scale development studies, it is necessary to ensure the face and content validity of the draft scale before collecting data from the sample. For this reason, it is recommended to seek the opinion of experts with experience in the researched topic [25]. The draft item pool (25 items) was sent to the experts via e-mail. A total of 15 experts, including physicians (*n* = 3) and nurse academicians (*n* = 12), who had previously conducted scale development studies and had studies on cardiometabolic diseases, were consulted. The experts involved in creating the content of the scale items were asked to evaluate each item in terms of appropriateness and comprehensibility. Based on the Davis technique, they were asked to score each statement between 1 and 4 points (1: suitable, 2: needs minor revision, 3: needs major revision, 4: unsuitable) and to write their opinions and suggestions about each item. The Content Validity Index (CVI) was used to evaluate expert opinions. This index is used to determine whether the experts consider each item necessary or not [26].

Following the initial content validity assessment using the Davis method, two items -nausea and dysphagia- were excluded due to CVI values below the acceptable threshold of 0.80 (both CVI = 0.73). Beyond the quantitative evaluation, the expert panel also considered the clinical relevance of each item. Nausea was excluded not only due to its low CVI score but also because it was deemed a non-specific symptom that is not strongly associated with cardiometabolic conditions. Dysphagia, although clinically significant in certain populations, was not classified as a symptom but rather as a post-stroke sequela, and thus was not considered appropriate for inclusion in a symptom severity scale. Considering the comments from the experts, the scale items were revised, and two items (Difficulty falling asleep/sustaining sleep and feeling of anxiety/worry) were added. Due to the added items, the draft scale consisting of 25 items was sent for expert opinion again and evaluated by five experts. After the second stage of expert evaluation, the CVI was calculated utilizing the Davis method, and no items were removed since there were no items below 0.80.

#### Pilot implementation stage

After the second expert opinion, a pilot study was conducted with a draft scale consisting of 25 items. The researcher applied the final version of the scale to 10 patients, and the patients were asked to evaluate the scale items based on meaningfulness, readability, comprehensibility of terms, and clarity. Since no negative feedback was received regarding the comprehensibility and readability of the scale, the scale items were found to be clear, and therefore, no changes were made. After all items were approved, the draft scale was finalized. The data obtained from the pilot study was not included in the main sample.

#### Main implementation stage

The final scale version was applied to 388 older patients with cardiometabolic multimorbidity, and validity and reliability studies were performed. The researcher administered the Patient Information Form, the SSS-CM, and the EQ-5D-3 L General Quality of Life Scale to assess parallel form validity using a face-to-face interview technique. Completing the study forms by the individuals took approximately 10–15 min. No missing responses were observed in the completed SSS-CM questionnaires. This was ensured through in-person data collection by trained research staff who reviewed each form for completeness at the time of administration.

### Data collection tools

#### Patient information form

Regarding the literature [2, 3, 5, 6], the patient information form developed by the researchers included 11 questions about socio-demographic characteristics (age, gender, educational status, smoking, and alcohol use) and information about the diagnosis of diseases (presence of coronary artery disease, diabetes mellitus, hypertension, stroke, and dyslipidemia and the duration of their diagnosis and hospitalization in the last year).

#### Symptom severity scale in patients with cardiometabolic multimorbidity (SSS-CM)

The scale developed by the researchers consists of a single dimension and 25 items to assess the severity of common symptoms in older adults with at least two cardiometabolic diseases (Supplementary Material 1). Each of the 25 items on the SSS-CM is rated on a 5-point Likert scale ranging from 0 (Not at all) to 4 (Very severe). The total score is obtained by summing all item scores, yielding a final score ranging from 0 to 100. No items require reverse scoring. Since the maximum possible score is 100, no further scoring transformation is applied. A higher total score indicates greater overall symptom severity in patients with multiple cardiometabolic diseases. To further clarify the scoring process, a sample scoring calculation using hypothetical data is presented in Supplementary Material 2.

*EQ-5D-3L Quality of Life Scale*: The EuroQol group developed this scale in 1987, comprising two main components [27]. The first component, the EQ-5D-3L index scale, evaluates five dimensions: mobility, self-care, usual activities, pain/discomfort, and anxiety/depression. Each dimension has three response options: no problem, some problems, and severe problems. Based on responses, an index score is calculated, ranging from − 0.59 to 1, where 0 represents death and 1 signifies optimal health. The second component, the EQ-5D VAS (Visual Analogue Scale), allows individuals to rate their current health status on a scale from 0 to 100 by marking a thermometer-like visual scale [27]. In a Turkish validity and reliability study conducted by Kahyaoğlu Sut and Ünsar [28] with patients diagnosed with acute coronary syndrome, the general health scale of EQ-5D demonstrated a high-reliability coefficient (Cronbach’s alpha = 0.860).

To assess criterion validity, the EQ-5D-3L was used as a parallel measure in this study. The decision was based on both theoretical and empirical evidence supporting a strong negative correlation between symptom burden and health-related quality of life in older adults with multimorbidity [10]. The EQ-5D-3 L, a widely validated tool, captures dimensions such as mobility, pain/discomfort, and anxiety/depression, which are directly influenced by symptom severity. Prior studies have demonstrated that individuals reporting higher symptom loads tend to have significantly lower EQ-5D index scores [9, 29]. Given the lack of population-specific symptom burden instruments, the EQ-5D-3L was considered an appropriate and clinically meaningful criterion measure for this study.

### Ethical consideration

This study was conducted following the principles of the Declaration of Helsinki. Ethical approval of the study was obtained from the Non-Interventional Ethics Committee of the Faculty of Medicine of the Sakarya University with the decision date of 28.12.2023 and protocol number 71522473-050.01.04-318591-384. Verbal and written informed consent were obtained from each participant before the interviews. Participants could withdraw from the study without any explanation and were not expected to make any payment.

### Data analysis

The data collected in this study were analyzed using SPSS 26.0 and AMOS statistical software. Categorical variables were summarized through frequency and percentage distributions, whereas numerical variables were reported with mean and standard deviation values. To assess the assumption of normality prior to factor analyses, descriptive skewness and kurtosis values were calculated for all 25 items. For the EFA sample (*n* = 200), skewness ranged from 0.05 to 1.93 and kurtosis from − 0.67 to 1.95. Similarly, in the CFA sample (*n* = 188), skewness values ranged from 0.34 to 1.80 and kurtosis from − 0.55 to 1.71. All values fell within the acceptable range of ± 2, supporting the appropriateness of using parametric methods in both EFA and CFA. Exploratory and confirmatory factor analysis were conducted to evaluate the scale’s validity. Reliability analysis was performed using Cronbach’s Alpha and McDonald’s Omega coefficients. Internal consistency was assessed through item analysis, while scale discrimination was examined using an independent samples t-test comparing the lower and upper 27% groups. Additionally, Pearson correlation analysis was applied to determine criterion validity.

## Results

### Characteristics of participants

Of the 200 patients who participated in the study for EFA, 52% were female, and 50% had primary/secondary education. Regarding chronic diseases, 57% of the participants had coronary heart disease, 83% had hypertension, 57.5% had diabetes mellitus, 13% had stroke, and 30.5% had dyslipidemia. The mean years of coronary heart disease diagnosis was 5.87 years, hypertension diagnosis was 9.81 years, diabetes mellitus diagnosis was 10 years, stroke diagnosis was 3.31 years, and dyslipidemia diagnosis was 6.87 years. The mean age was 67.41 years (SD = 5.78) (Table 1).

**Table 1:** Descriptive characteristics of participants for EFA

Variables	*N*		*n*	%
Gender	200	Female	104	52.0
		Male	96	48.0
Education status	200	Literate (no formal degree)	62	31.0
		Primary-secondary school	100	50.0
		High School	31	15.5
		University	7	3.5
Smoking status	200	Current smoker	35	17.5
		Never smoked	76	38.0
		Quit smoking	89	44.5
Alcohol consumption status	200	Still drinks	3	1.5
		Never drank	125	62.5
		Quit drinking	72	36.0
CHD	200	Yes	114	57.0
		No	86	43.0
HT	200	Yes	166	83.0
		No	34	17.0
DM	200	Yes	115	57.5
		No	85	42.5
Stroke	200	Yes	26	13.0
		No	174	87.0
Dyslipidemia	200	Yes	61	30.5
		No	139	69.5
Hospitalization within one year	200	None	97	48.5
		1–2 times	86	43.0
		2+	17	8.5
		M ± SD	Min	Max
Age (Years)	200	67.41 ± 5.78	65	88
CHD Diagnosis (Years)	114	5.87 ± 4.40	1	20
HT Diagnosis (Years)	166	9.81 ± 4.94	1	30
DM Diagnosis (Years)	115	10.00 ± 4.73	1	30
Stroke Diagnosis (Years)	26	3.31 ± 4.12	1	20
Dyslipidemia Diagnosis (Years)	61	6.87 ± 3.50	2	15

Data are presented as mean (M), standard deviation (SD), n (%)

Abbreviation: *CHD* coronary heart disease; *HT* hypertension; *DM* diabetes mellitus

Of the 188 patients who participated in the study for CFA, 50.5% were male, and the majority (43.1%) had primary/secondary education. Among the participants, 80.9% had hypertension, 58.0% had diabetes mellitus, 52.7% had coronary heart disease, 17.6% had a stroke, and 30.3% had dyslipidemia. The mean age at diagnosis of coronary heart disease and diabetes mellitus was 6.83 and 10.65 years, respectively, indicating that their disease history generally covered a long period. The mean age was 69.19 years (SD = 5.99) (Table 2).

**Table 2:** Descriptive characteristics of participants for CFA

Variables	*N*		*n*	%
Gender	188	Female	93	49.5
		Male	95	50.5
Education status	188	Literate (no formal degree)	68	36.2
		Primary-secondary school	81	43.1
		High School	31	16.5
		University	8	4.3
Smoking status	188	Current smoker	37	19.7
		Never smoked	80	42.6
		Quit smoking	71	37.8
Alcohol consumption status	188	Still drinks	6	3.2
		Never drank	117	62.2
		Quit drinking	65	34.6
CHD	188	Yes	99	52.7
		No	89	47.3
HT	188	Yes	152	80.9
		No	36	19.1
DM	188	Yes	109	58.0
		No	79	42.0
Stroke	188	Yes	33	17.6
		No	115	82.4
Dyslipidemia	188	Yes	57	30.3
		No	131	69.7
Hospitalization within one year	188	None	90	47.9
		1–2 times	85	45.2
		2+	13	6.9
	188	M ± SD	Min	Max
Age (Years)	188	69.19 ± 5.99	65	90
CHD Diagnosis (Years)	99	6.83 ± 5.08	1	23
HT Diagnosis (Years)	152	9.66 ± 6.59	1	40
DM Diagnosis (Years)	109	10.65 ± 5.75	2	30
Stroke Diagnosis (Years)	33	4.12 ± 4.28	1	20
Dyslipidemia Diagnosis (Years)	57	6.79 ± 3.10	1	15

Data are presented as mean (M), standard deviation (SD), n (%)

Abbreviation: *CHD* coronary heart disease; *HT* hypertension; *DM* diabetes mellitus

### Exploratory factor analysis

EFA revealed that the scale had a one-factor structure, and the scale items showed a high correlation with this factor. The Kaiser-Meyer-Olkin (KMO) test was conducted to assess the adequacy of the sample, and the KMO value was found to be 0.965. In addition, Bartlett’s Test of Sphericity (χ² = 5065.520, df = 300, *p* < 0.001) indicated a sufficient correlation between the variables.

The factor analysis was conducted using Principal Component Analysis, and the Varimax rotation method was used to make the factor structure simpler and more interpretable. As a result of the rotation process, all the scale items loaded significantly under a single factor. The factor loadings for the 25 items in the scale ranged between 0.658 and 0.898, and factor loadings of 0.60 and above were taken as acceptable threshold values in this study. The values obtained show that all items were above this threshold and loaded strongly on the factor.

**Fig. 3:**
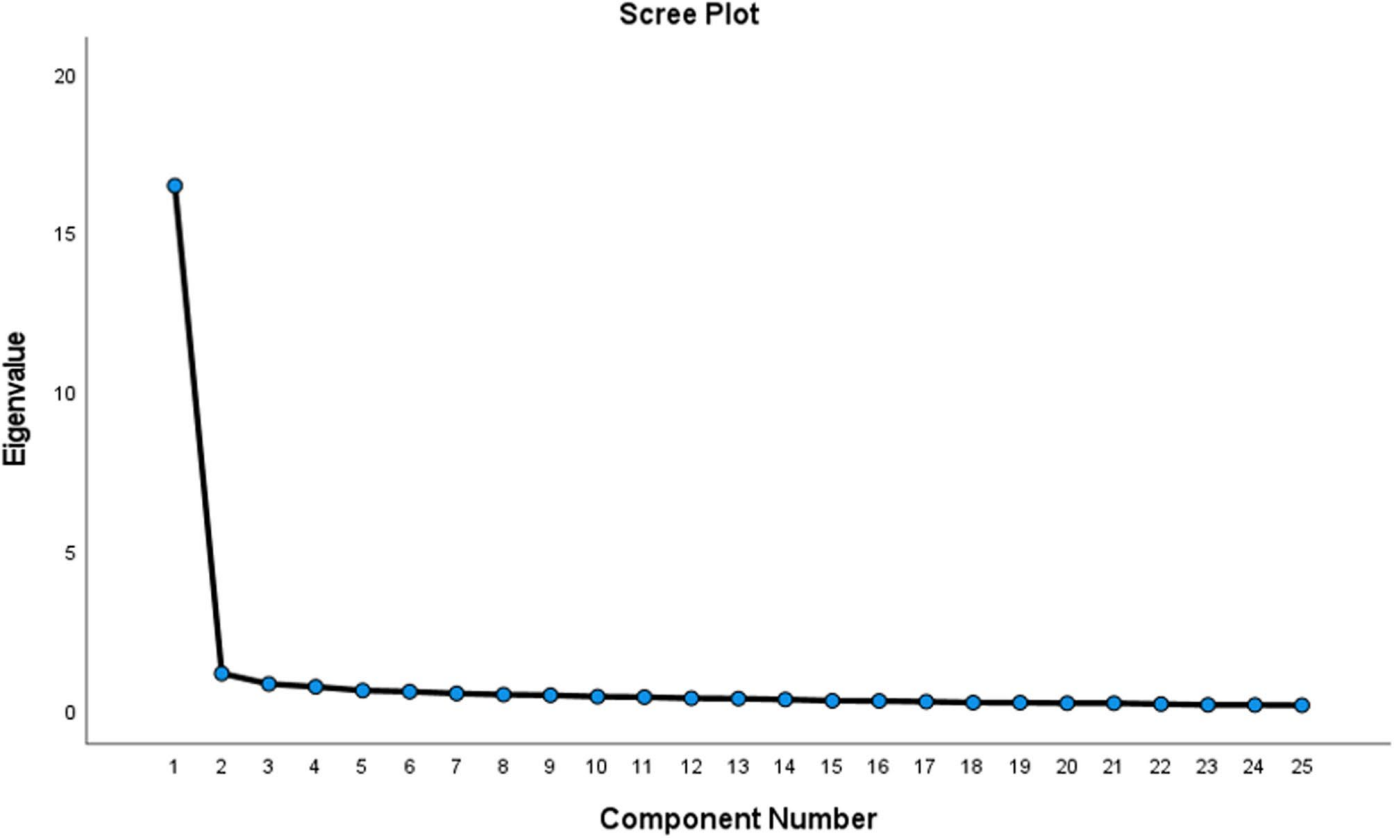
Scree plot braph

As a result of the factor analysis, the eigenvalue of the first factor of the scale was found to be 16.438, and this factor explained 65.753% of the total variance. This ratio is well above the widely accepted lower limit of 40% and is a strong indicator supporting the construct validity of the scale. Moreover, the presence of only one factor with an eigenvalue above 1 indicates that a one-factor structure is appropriate. Table 3 shows the loading levels of each item on the relevant factor and the explained common variance (communality of items) values. The common variance values range between 0.433 and 0.806, and these values indicate that the items are adequately explained by the factor. In addition, the Scree Plot graph showed the distribution of the eigenvalues of the factor analysis results (Fig. 3).

**Table 3:** Factor loads and common variance values

Item	Factor Load	Common Variance (Extraction)
S1	0.756	0.571
S2	0.747	0.558
S3	0.812	0.660
S4	0.795	0.632
S5	0.832	0.693
S6	0.851	0.723
S7	0.865	0.748
S8	0.807	0.651
S9	0.861	0.741
S10	0.824	0.680
S11	0.853	0.727
S12	0.866	0.749
S13	0.898	0.806
S14	0.789	0.623
S15	0.879	0.773
S16	0.658	0.433
S17	0.819	0.671
S18	0.838	0.702
S19	0.837	0.700
S20	0.739	0.547
S21	0.845	0.714
S22	0.760	0.577
S23	0.748	0.560
S24	0.823	0.677
S25	0.725	0.526

### Confirmatory factor analysis

**Fig. 4:**
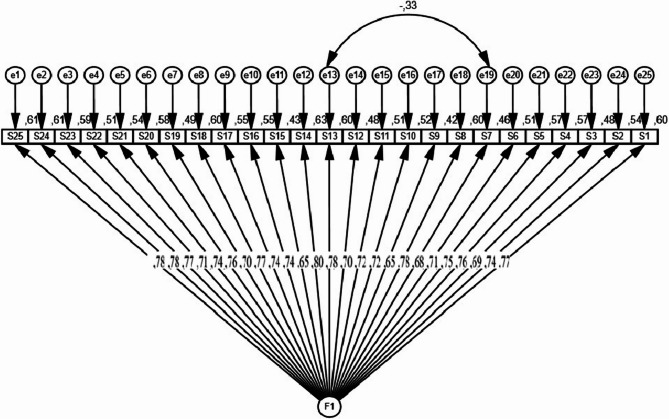
Diagram of Confirmatory Factor Analysis with Factor Loadings

CFA was conducted to evaluate the one-factor structure of the scale through goodness of fit indices (Fig. 4). The normed chi-square (χ2/df) ratio was calculated as 1.739 and remained within the acceptable limit (0 ≤ χ2/df ≤ 5). Comparative fit index (CFI: 0.938), incremental fit index (IFI: 0.938), and Tucker Lewis index (TLI: 0.932) values were relatively high, indicating that the model provided a good fit. Goodness-of-fit index (GFI: 0.802) and normed fit index (NFI: 0.865) values were close to the acceptable lower limits. Root mean square error of approximation (RMSEA: 0.063) and root mean square residual (RMR: 0.013) values support the satisfactory fit of the model with the data (Table 4).

**Table 4:** Goodness-of-fit indices for CFA

Indices	Good Fit	Acceptable Fit		Results
χ2	476.428			
df	274			
χ2/df (CMIN/DF)	0 ≤ χ2/df ≤ 3		3 ≤ χ2/df ≤ 5	1.739
CFI	0.95 ≤ CFI ≤ 1.00		0.80 ≤ CFI ≤ 0.95	0.938
GFI	0.95 ≤ GFI ≤ 1.00		0.80 ≤ GFI ≤ 0.95	0.802
NFI	0.95 ≤ NFI ≤ 1.00		0.80 ≤ NFI ≤ 0.95	0.865
IFI	0.95 ≤ IFI ≤ 1.00		0.80 ≤ IFI ≤ 0.95	0.938
TLI	0.95 ≤ TLI ≤ 1.00		0.80 ≤ TLI ≤ 0.95	0.932
RMSEA	0 ≤ RMSEA ≤ 0.05		0.05 ≤ RMSEA ≤ 0.08	0.063
RMR	0 ≤ RMSEA ≤ 0.05		0.05 ≤ RMR ≤ 0.08	0.013

Abbreviation: χ2/*df* normed chi-square; *CFI* comparative fit index; *GFI* goodness-of-fit index; *NFI* normed fit index; *IFI* incremental fit index; *TLI* Tucker Lewis index; *RMSEA* root mean square error of approximation; *RMR* root mean square residual

Based on CFA, the factor loads, t values, and significance levels of the items supporting the one-factor structure of the scale are presented in Table 5 to evaluate the scale’s construct validity. The standard factor loads (Std. β) of the items ranged between 0.653 and 0.797 and were at acceptable levels. In addition, all t-values ranged between 9.582 and 12.256 and were significant at the *p* < 0.001 level.

**Table 5:** Confirmatory factor analysis factor loads

Items and Factors			β	Std. β	S.Error	t	*p*
S25	<---	F1	0.784	1.000			
S24	<---	F1	0.781	1.083	0.091	11.943	*p* < 0.001
S23	<---	F1	0.768	0.990	0.085	11.684	*p* < 0.001
S22	<---	F1	0.711	0.823	0.078	10.616	*p* < 0.001
S21	<---	F1	0.737	0.852	0.077	11.090	*p* < 0.001
S20	<---	F1	0.759	0.947	0.082	11.517	*p* < 0.001
S19	<---	F1	0.703	0.823	0.079	10.471	*p* < 0.001
S18	<---	F1	0.772	0.916	0.078	11.763	*p* < 0.001
S17	<---	F1	0.739	1.002	0.090	11.138	*p* < 0.001
S16	<---	F1	0.741	0.808	0.072	11.172	*p* < 0.001
S15	<---	F1	0.653	0.726	0.076	9.582	*p* < 0.001
S14	<---	F1	0.797	0.961	0.078	12.256	*p* < 0.001
S13	<---	F1	0.777	0.969	0.082	11.851	*p* < 0.001
S12	<---	F1	0.694	0.777	0.075	10.302	*p* < 0.001
S11	<---	F1	0.717	0.763	0.071	10.731	*p* < 0.001
S10	<---	F1	0.723	0.852	0.079	10.842	*p* < 0.001
S9	<---	F1	0.650	0.777	0.081	9.534	*p* < 0.001
S8	<---	F1	0.776	0.979	0.083	11.842	*p* < 0.001
S7	<---	F1	0.678	0.745	0.075	10.002	*p* < 0.001
S6	<---	F1	0.715	0.870	0.081	10.693	*p* < 0.001
S5	<---	F1	0.755	0.973	0.085	11.439	*p* < 0.001
S4	<---	F1	0.757	0.913	0.080	11.472	*p* < 0.001
S3	<---	F1	0.694	0.807	0.078	10.300	*p* < 0.001
S2	<---	F1	0.737	0.925	0.083	11.101	*p* < 0.001
S1	<---	F1	0.772	0.989	0.084	11.772	*p* < 0.001

### Criterion validity

A significant negative correlation was found between the total scale score and EQ-5D VAS health status (r = −0.430, *p* < 0.001). This relationship reveals that the perception of health status worsens with increasing symptom severity. Similarly, a highly negative correlation was found between the scale total score and EQ-5D-3L quality of life (r = −0.790, *p* < 0.001).

### Reliability analysis

When evaluating the scale’s internal consistency, cronbach’s alpha coefficient was found to be 0.977, and mcdonald’s Omega coefficient was found to be 0.978. Item-total correlations ranged from 0.636 to 0.887, indicating that all items were appropriate to the scale structure and consistently represented the measured concept.

### Item discrimination

To determine the discrimination of the scale, a t-test analysis was performed between the lower 27% and upper 27% groups. According to the analysis results, the mean symptom total score in the lower group was 7.50 (SD = 6.21), while the mean in the upper group was 36.61 (SD = 9.11). A statistically significant difference was found between the groups (t = −19.406, *p* < 0.001). Cohen’s d value was − 3.735, indicating a significant effect. Furthermore, the confidence interval of the mean difference (−32.09 to −26.14) supported that the difference between the two groups was consistently demonstrated.

## Discussion

This scale was developed considering the relevant literature to determine the severity of common symptoms affecting the quality of life in older patients with cardiometabolic multimorbidity. This study showed that the SSS-CM, consisting of a single-factor structure, meets content, construct validity, and reliability criteria. Consequently, healthcare professionals can use this scale to comprehensively assess symptoms in patients diagnosed with cardiometabolic multimorbidity.

The KMO coefficient and Bartlett’s test of sphericity are commonly used to assess the suitability of a dataset for factor analysis in construct validity evaluations [30, 31]. According to the literature, a KMO value above 0.60, ideally approaching 1, indicates that the data are appropriate for factor analysis [31]. If the KMO value exceeds 0.50, factor analysis can be performed; values between 0.70 and 0.80 suggest moderate adequacy, between 0.80 and 0.90 indicate good adequacy, and values above 0.90 reflect excellent sampling adequacy [30, 31]. Additionally, a significant Bartlett’s test of sphericity result confirms that the correlation matrix of the scale items is suitable for factor analysis. In this study, the KMO coefficient was found to be 0.965, and Bartlett’s test of sphericity was statistically significant. These results indicate that the sample size was sufficient, and the scale met the necessary conditions for factor analysis.

Considering the results of EFA, it is emphasized that whether the scale has a single-factor or multi-factor structure should be considered. The literature reports that at least 30% of the total variance should be explained in single-factor scales, and 40–60% is ideal [19]. According to the results of the EFA conducted in our study, the SSS-CM was found to have a single-factor structure. The factors in the scale explained 65.753% of the total variance. This finding showed that each item was sufficiently related to the scale [32]. In addition, in the Scree Plot graph, the eigenvalue of the first component had a significantly higher value than the others, and a rapid decrease was observed in the following components. Therefore, the graph clearly showed that explaining the scale with the first factor is appropriate and that additional factors will not explain a significant variance [33]. These findings provide important visual evidence supporting the factor analysis results and the scale’s one-factor structure.

In the CFA used within the scope of validity analysis in our study, the factor analysis fit indices of the scale showed that the fit of the model with the data is acceptable. Regarding compatibility indices, RMSEA values less than 0.08 and χ2/df less than 3.0 indicate a good fit [34]. The compatibility index results in our findings (χ2/df = 1.739 and RMSEA = 0.063) showed that the fit of the scale model with the data is acceptable. GFI, NFI, and CFI values close to 1 indicate a good fit [21, 34]. As a result of the analysis, the GFI value of the scale was 0.802, the NFI value was 0.865, and the CFI value was 0.938. These values indicate acceptable fit. In addition, the CFA results revealed that all items loaded significantly on a single-factor structure. The fact that the items had a high and significant relationship with the factor confirms the scale’s construct validity and supports its reliability.

While the EFA and CFA analyses supported a unidimensional structure, this approach is also theoretically grounded in how symptom burden is conceptualized among patients with cardiometabolic multimorbidity [7]. Symptoms may arise in isolation, co-occur with other symptoms, or even precede one another as part of a dynamic and interconnected process [35]. For instance, dyspnea, fatigue, sleep disturbances, and mood alterations frequently manifest together and interact synergistically, contributing to a cumulative and holistic perception of illness burden. Particularly in older adults with cardiometabolic multimorbidity, the simultaneous presence of multiple symptoms or the aggregation of symptom burden over time may render categorical distinctions between symptoms impractical or clinically irrelevant. Accordingly, given that both the statistical and theoretical background supported a single-factor structure, the SSS-CM is recommended to be used as a unidimensional instrument, producing a single composite score. This approach ensures ease of interpretation and reflects the overarching construct of overall symptom severity in older adults with cardiometabolic multimorbidity.

On the other hand, this structure may not fully capture the clinical complexity of symptom clusters in cardiometabolic multimorbidity. Given the potential distinctions between physical, psychological, and autonomic symptoms, future studies with larger and more diverse samples should consider investigating possible sub-dimensions. Such multidimensional modeling could enhance the clinical interpretability and specificity of the SSS-CM in targeted care strategies.

Regarding validity analyses, a correlation is sought between the tested scale and another parallel form with proven validity and reliability. In this study, the results of the correlation analysis showed that the criterion validity of the SSS-CM was high. A significant negative correlation was found between the scale total score and EQ-5D Quality of Life and VAS health status scores. This strong negative correlation indicates that increasing symptom severity has a significant negative impact on the overall quality of life and worsens the perception of health status. These findings support that the symptom severity scale is a valid measurement tool by establishing significant and expected relationships with parallel forms.

The Cronbach’s alpha coefficient for the scale was calculated as 0.977. According to the literature, Cronbach’s alpha coefficient ranges from 0.0 to 1.0, with values between 0.60 and 0.80 indicating a high level of reliability and values of 0.80 and above signifying excellent reliability [36, 37]. Based on these criteria, Cronbach’s alpha value obtained in this study demonstrates excellent reliability and is consistent with the findings in the literature.

In item-total score analysis, the effectiveness of an item in assessing the intended construct is determined by the correlation coefficient, with higher values indicating stronger adequacy. A correlation coefficient below 0.30 suggests a substantial issue with the item, which may warrant its removal from the scale. Conversely, item-total score correlations exceeding 0.30 are generally considered acceptable for reliability [37]. In this study, item-total correlation values ranged from 0.636 to 0.887, demonstrating that all items aligned well with the overall scale structure and reliably represented the intended concept.

Finally, a t-test analysis was performed between the lower 27% and upper 27% groups to determine the discrimination of the scale [38]. This analysis was conducted to assess whether the scale could successfully discriminate patients with different levels of symptom severity [38]. According to the analysis results, a statistically significant difference was found between the total score for the mean symptom severity in the lower group and the mean in the upper group. These findings indicate that the scale can successfully discriminate patients with different levels of symptom severity and that the scale has a strong discrimination feature. In particular, the success of the scale items in measuring a specific characteristic provides important evidence for the validity and reliability of the measurement tool.

The SSS-CM was developed to address key limitations of both generic and disease-specific symptom assessment tools in the context of cardiometabolic multimorbidity. While generic tools such as the ESAS, MSAS, and MDASI were originally designed for oncology or palliative care settings, they do not fully reflect the complex, multi-system symptom burden associated with coexisting cardiovascular, metabolic, and neurological conditions. Conversely, disease-specific tools, such as those for diabetes, stroke, or coronary artery disease, offer focused assessments but are impractical for use in multimorbid patients, as multiple instruments would need to be administered simultaneously. In contrast, the SSS-CM provides a unified, clinically relevant, and time-efficient framework for capturing symptom burden across a broad spectrum of conditions commonly seen in older adults with cardiometabolic multimorbidity (Supplementary Material 3).

Accordingly, the clinical utility of the SSS-CM lies in its ability to provide a quantifiable assessment of symptom burden among older patients with cardiometabolic multimorbidity. The 0–100 scoring system allows clinicians to quickly identify high-severity symptoms, prioritize symptom management strategies, and monitor patient response over time. The scale can be administered during routine assessments in inpatient or outpatient settings and integrated into electronic health records to support decision-making, risk stratification, and individualized care plans. In multidisciplinary care contexts, the SSS-CM may serve as a communication tool between nurses, physicians, geriatricians, and allied health professionals for holistic symptom management.

### Limitations

A key limitation of this study is the use of purposive sampling from inpatient departments of a single hospital in Turkey, which may constrain the cultural, demographic, and clinical representativeness of the study population. Consequently, the generalizability of the SSS-CM to other healthcare settings or populations, including younger individuals or those with milder disease profiles, may be limited. To address this, future research should focus on validating the scale in multicenter and more heterogeneous clinical contexts, including outpatient services, primary care clinics, and community-dwelling older adults with varying levels of symptom burden. Additionally, involving participants from diverse geographic regions and healthcare systems would further enhance the external validity and cross-context applicability of the SSS-CM. Another notable limitation is the lack of test-retest reliability assessment, which restricts the understanding of the scale’s temporal stability. Additionally, the scale’s responsiveness to clinical change over time, which is critical for its use in monitoring symptom progression or intervention outcomes, was not evaluated due to the cross-sectional design. Future longitudinal studies should explore these aspects to ensure the SSS-CM’s robustness in repeated measurements and dynamic clinical contexts. Lastly, although the SSS-CM demonstrated good readability in pilot testing, the 25-item length may still be burdensome for frail, functionally limited, or cognitively impaired older adults. Future studies should consider developing a shortened version of the scale using Rasch analysis or other item reduction techniques to enhance usability in clinical settings without compromising psychometric quality.

## Conclusion

The overall fit index values of the single-factor 25-item structure of the SSS-CM Scale showed a good fit. Cronbach’s alpha analysis method was used in reliability analyses, and it was found that all scale items measured the same feature, and the scale was reliable. In the context of parallel form reliability, a significant negative relationship was found between the participants’ symptom severity and quality of life scores. Accordingly, the scales were significantly correlated and met the EQ-5D parallel form reliability criteria.

In conclusion, the SSS-CM is a valid and reliable measurement tool developed to determine symptom severity in hospitalized older patients with cardiometabolic multimorbidity. Developing and validating the symptom severity scale for these patients is important to manage the high symptom burden and complexity of treatment and care. The consideration of personalized and patient-centered care to alleviate symptom burdens and improve quality of life may have important implications for the clinical management of this vulnerable patient population. Future longitudinal and interventional studies are needed to determine whether the routine use of the scale contributes to enhanced symptom monitoring, more timely and targeted interventions, reduced hospitalizations, or improved quality of life. Such evidence would strengthen the justification for integrating the SSS-CM into routine geriatric and multiple chronic disease management workflows.

## Supplementary Information

Below is the link to the electronic supplementary material.

Supplementary Material 1

Supplementary Material 2

Supplementary Material 3

## Data Availability

Data available on request due to privacy/ethical restrictions.

## References

[1] Bai A, Chen Q, Geldsetzer P, Gray M, Xie Z, Zhang D, et al. Functional dependency and cardiometabolic Multimorbidity in older people: pooled analysis of individual-level data from 20 countries. Age Ageing. 2024;53. 10.1093/ageing/afae269 39686679

[2] Han Y, Hu Y, Yu C, Guo Y, Pei P, Yang L, et al. Lifestyle, cardiometabolic disease, and Multimorbidity in a prospective Chinese study. Eur Heart J. 2021;42:3374–84. 34333624 10.1093/eurheartj/ehab413 PMC8423468

[3] Jin Y, Liang J, Hong C, Liang R, Luo Y. Cardiometabolic multimorbidity, lifestyle behaviours, and cognitive function: a multicohort study. Lancet Healthy Longev. 2023;4:e265–73. 37150183 10.1016/S2666-7568(23)00054-5

[4] Dove A, Guo J, Marseglia A, Fastbom J, Vetrano DL, Fratiglioni L, et al. Cardiometabolic Multimorbidity and incident dementia: the Swedish twin registry. Eur Heart J. 2023;44:573–82. 36577740 10.1093/eurheartj/ehac744 PMC9925275

[5] Zheng Y, Zhou Z, Wu T, Zhong K, Hu H, Zhang H, et al. Association between composite lifestyle factors and cardiometabolic Multimorbidity in chongqing, china: A cross-sectional exploratory study in people over 45 years and older. Front Pub Health. 2023;11. 10.3389/fpubh.2023.1118628 PMC9929179 36817881

[6] Zhang H, Duan X, Rong P, Dang Y, Yan M, Zhao Y, et al. Effects of potential risk factors on the development of cardiometabolic Multimorbidity and mortality among the elders in China. Front Cardiovasc Med. 2022;9. 10.3389/fcvm.2022.966217 PMC9502033 36158847

[7] Willadsen TG, Siersma V, Nicolaisdottir DR, Jarbol D, Guassora AD, Reventlow S, et al. Symptom burden in multimorbidity: a population-based combined questionnaire and registry study from Denmark. BMJ Open. 2021;11:e041877. 33849847 10.1136/bmjopen-2020-041877 PMC8051398

[8] Otieno P, Asiki G, Wekesah F, Wilunda C, Sanya RE, Wami W, et al. Multimorbidity of cardiometabolic diseases: a cross-sectional study of patterns, clusters and associated risk factors in sub-Saharan Africa. BMJ Open. 2023;13:e064275. 36759029 10.1136/bmjopen-2022-064275 PMC9923299

[9] Lu H, Dong X-X, Li D-L, Nie X-Y, Wang P, Pan C-W. Multimorbidity patterns and health-related quality of life among community-dwelling older adults: evidence from a rural town in suzhou, China. Qual Life Res. 2024;33:1335–46. 38353890 10.1007/s11136-024-03608-0

[10] Steell L, Krauth SJ, Ahmed S, Dibben GO, McIntosh E, Hanlon P, et al. Multimorbidity clusters and their associations with health-related quality of life in two UK cohorts. BMC Med. 2025;23:1. 39773733 10.1186/s12916-024-03811-3 PMC11708164

[11] Klompstra L, Ekdahl AW, Krevers B, Milberg A, Eckerblad J. Factors related to health-related quality of life in older people with Multimorbidity and high health care consumption over a two-year period. BMC Geriatr. 2019;19:187. 31277674 10.1186/s12877-019-1194-z PMC6612189

[12] Chang VT, Hwang SS, Thaler HT, Kasimis BS, Portenoy RK. Memorial symptom assessment scale. Expert Rev Pharmacoecon Outcomes Res. 2004;4:171–8. 19807521 10.1586/14737167.4.2.171

[13] Portenoy RK, Thaler HT, Kornblith AB, McCarthy Lepore J, Friedlander-Klar H, Kiyasu E, et al. The memorial symptom assessment scale: an instrument for the evaluation of symptom prevalence, characteristics and distress. Eur J Cancer. 1994;30:1326–36. 10.1016/0959-8049(94)90182-1 7999421

[14] Cleeland CS, Mendoza TR, Wang XS, Chou C, Harle MT, Morrissey M, et al. Assessing symptom distress in cancer patients. Cancer. 2000;89:1634–46. 11013380 10.1002/1097-0142(20001001)89:7<1634::aid-cncr29>3.0.co;2-v

[15] Grootenhuis PA, Snoek FJ, Heine RJ, Bouter LM. Development of a type 2 diabetes symptom checklist: a measure of symptom severity. Diabet Med. 1994;11:253–61. 8033523 10.1111/j.1464-5491.1994.tb00268.x

[16] Nieveen JL, Zimmerman LM, Barnason SA, Yates BC. Development and content validity testing of the cardiac symptom survey in patients after coronary artery bypass grafting. Heart Lung. 2008;37:17–27. 18206523 10.1016/j.hrtlng.2006.12.002

[17] Beal CC, Ogola G, Allen L. Validity and reliability of the responses to ischemic stroke symptoms questionnaire. J Neurosci Nurs. 2019;51:287–91. 31688280 10.1097/JNN.0000000000000474

[18] Juárez-García A, González-Muñoz EL, Aguirre-Moreno A. Validity, sensitivity and specificity of a scale to assess cardiovascular symptoms. Cardiovasc Metabolic Sci. 2019;30:100–13.

[19] Schreiber JB. Issues and recommendations for exploratory factor analysis and principal component analysis. Res Social Administrative Pharm. 2021;17:1004–11. 10.1016/j.sapharm.2020.07.027 33162380

[20] Memon MA, Ting H, Cheah J-H, Thurasamy R, Chuah F, Cham TH. Sample size for survey research: review and recommendations. J Appl Struct Equation Model. 2020;4:i–xx.

[21] Kyriazos T, Poga-Kyriazou M. Applied psychometrics: estimator considerations in commonly encountered conditions in CFA, SEM, and EFA practice. Psychology. 2023;14:799–828.

[22] Lorenzo-Seva U. SOLOMON: a method for splitting a sample into equivalent subsamples in factor analysis. Behav Res Methods. 2021;54:2665–77. 34918226 10.3758/s13428-021-01750-y PMC9729132

[23] Mondo M, Sechi C, Cabras C. Psychometric evaluation of three versions of the Italian perceived stress scale. Curr Psychol. 2021;40:1884–92.

[24] Montoya AK, Edwards MC. The poor fit of model fit for selecting number of factors in exploratory factor analysis for scale evaluation. Educ Psychol Meas. 2021;81:413–40. 33994558 10.1177/0013164420942899 PMC8072951

[25] Kishore K, Jaswal V, Kulkarni V, De D. Practical guidelines to develop and evaluate a questionnaire. Indian Dermatol Online J. 2021;12:266–75. 33959523 10.4103/idoj.IDOJ_674_20 PMC8088187

[26] Davis LL. Instrument review: getting the most from a panel of experts. Appl Nurs Res. 1992;5:194–7.

[27] Rabin R, de Charro F. EQ-SD: a measure of health status from the EuroQol group. Ann Med. 2001;33:337–43. 11491192 10.3109/07853890109002087

[28] Kahyaoglu Sut H, Unsar S, Is. EQ-5D a valid quality of life instrument in patients with acute coronary syndrome? Anadolu Kardiyoloji dergisi/the Anatolian. J Cardiol. 2011. 10.5152/akd.2011.037. 10.5152/akd.2011.037 21342862

[29] Buchholz I, Janssen MF. EQ-5D-3L norms for the European older population: Country-Specific norms for 15 European Countires based on the survey of health, ageing, and retirement in Europe. Value Health. 2023;26:721–32. 36396535 10.1016/j.jval.2022.09.2478

[30] Green SB, Yang Y. Evaluation of dimensionality in the assessment of internal consistency reliability: coefficient alpha and Omega coefficients. Educational Measurement: Issues Pract. 2015;34:14–20.

[31] Shrestha N. Factor analysis as a tool for survey analysis. Am J Appl Math Stat. 2021;9:4–11.

[32] Boateng GO, Neilands TB, Frongillo EA, Melgar-Quiñonez HR, Young SL. Best practices for developing and validating scales for health, social, and behavioral research: A primer. Front Pub Health. 2018;6. 10.3389/fpubh.2018.00149 PMC6004510 29942800

[33] den Reijer AHJ, Otter PW, Jacobs JPAM. An heuristic scree plot criterion for the number of factors. Stat Pap. 2024;65:3991–4000.

[34] Mohanasundaram SS. Fit indices in structural equation modeling and confirmatory factor analysis: reporting guidelines. Asian J Econ Bus Acc. 2024;24:561–77.

[35] Harris CS, Dodd M, Kober KM, Dhruva AA, Hammer MJ, Conley YP, et al. Advances in conceptual and methodological issues in symptom cluster research. Adv Nurs Sci. 2022;45:309–22. 10.1097/ANS.0000000000000423 PMC9616968 35502915

[36] Kennedy I. Sample size determination in Test-Retest and Cronbach alpha reliability estimates. Br J Contemp Educ. 2022;2:17–29.

[37] DeVellis RF, Thorpe CT. Scale development: theory and applications. Fifth, Sage. 2021.

[38] Hasançebi B, Terzi Y, Küçük Z. Madde Güçlük İndeksi ve madde Ayırt edicilik indeksine Dayalı çeldirici analizi. Gümüşhane Üniversitesi Fen Bilimleri Enstitüsü Dergisi. 2020;10:224–40.

