# Supplementary material for: Development and psychometric testing of symptom severity scale in older patients with cardiometabolic multimorbidity
Source: BMC Geriatr. 2025 Oct 14;25:771. doi: 10.1186/s12877-025-06370-1 (PMC12522849; doi:10.1186/s12877-025-06370-1)
Supplement: Supplementary file 1 — Supplementary Material 1 [file 12877_2025_6370_MOESM1_ESM.docx]

**Supplementary Material 1. Turkish-Original Version of the SSS-CM**

Aşağıdaki ölçekte kardiyometabolik (koroner kalp hastalığı, inme, diyabet ve hipertansiyon hastalıklarından en az iki tanesine yönelik tanısı olan) **çoklu hastalığı** olan bireylerde görülen belirtiler yer almaktadır. **Son bir ay içinde** bu belirtileri yaşadıysanız, bu belirtilerin sizi ne kadar **rahatsız ettiğini** ilgili kutucuğu işaretleyerek belirtiniz.

| **Son bir ay (4 hafta) içinde aşağıdaki belirtileri yaşadınız mı?** | | **Yaşadıysanız sizi ne kadar rahatsız etti?** | | | | | |
| --- | --- | --- | --- | --- | --- | --- | --- |
|  | | **0**  **(hiç)** | **1**  **(hafif)** | **2**  **(orta)** | **3**  **(şiddetli)** | **4**  **(çok şiddetli)** |  |
|  | Ağız kuruluğu |  |  |  |  |  |  |
|  | Bacaklarda şişlik/ödem |  |  |  |  |  |  |
|  | Baş ağrısı |  |  |  |  |  |  |
|  | Baş dönmesi |  |  |  |  |  |  |
|  | Bayılma |  |  |  |  |  |  |
|  | Bilinç bulanıklığı, anlamada güçlük |  |  |  |  |  |  |
|  | Bulanık/çift görme |  |  |  |  |  |  |
|  | Burun kanaması |  |  |  |  |  |  |
|  | Denge, koordinasyon ve yürüme zorluğu |  |  |  |  |  |  |
|  | Ellerde veya ayaklarda uyuşma/karıncalanma |  |  |  |  |  |  |
|  | Göğüste ağrı, yanma ve sıkışma hissi |  |  |  |  |  |  |
|  | Halsizlik / yorgunluk |  |  |  |  |  |  |
|  | Huzursuzluk hissi |  |  |  |  |  |  |
|  | Kafada karışıklık, dikkat dağınıklığı, hafıza, hatırlama sorunları |  |  |  |  |  |  |
|  | Kalp çarpıntısı |  |  |  |  |  |  |
|  | Kaygı/endişe hissi |  |  |  |  |  |  |
|  | Kol veya bacaklarda güç/his kaybı |  |  |  |  |  |  |
|  | Konuşma güçlüğü |  |  |  |  |  |  |
|  | Kulak çınlaması |  |  |  |  |  |  |
|  | Kusma |  |  |  |  |  |  |
|  | Nefes darlığı |  |  |  |  |  |  |
|  | Sık idrara çıkma |  |  |  |  |  |  |
|  | Aşırı terleme |  |  |  |  |  |  |
|  | Uyku hali/sersemlik hissi |  |  |  |  |  |  |
|  | Uykuya dalma/sürdürme güçlüğü |  |  |  |  |  |  |

**Ölçek Puanlama:** Her maddede işaretlenen rakamlar toplanır. Ölçekten alınabilecek puan **0-100** arasında değişmektedir. Toplam puanın artması çoklu hastalığı olan bireylerde semptom şiddetinin daha yüksek olduğunu göstermektedir.
