# Supplementary material for: Development and psychometric testing of symptom severity scale in older patients with cardiometabolic multimorbidity
Source: BMC Geriatr. 2025 Oct 14;25:771. doi: 10.1186/s12877-025-06370-1 (PMC12522849; doi:10.1186/s12877-025-06370-1)
Supplement: Supplementary file 2 — Supplementary Material 2 [file 12877_2025_6370_MOESM2_ESM.docx]

**Supplementary Material 2. Example of SSS-CM Scoring**

Scale Scoring: Each of the 25 items on the Symptom Severity Scale in Patients with Cardiometabolic Multimorbidity (SSS-CM) is rated on a 5-point Likert scale ranging from 0 (Not at all) to 4 (Very severe). The total score is obtained by summing all item scores, yielding a final score ranging from 0 to 100. No items require reverse scoring. Since the maximum possible score is 100, no further scoring transformation is applied. A higher total score indicates greater overall symptom severity in patients with multiple cardiometabolic diseases.

To facilitate understanding of the scoring system for the SSS-CM, the following example demonstrates how to calculate the total score for a hypothetical patient.

| **Item No** | **Symptom Description** | **Patient Response (0–4)** |
| --- | --- | --- |
| 1 | Dry mouth | 3 |
| 2 | Leg swelling/edema | 2 |
| 3 | Headache | 1 |
| ... | ... | ... |
| ... | ... | ... |
| 25 | Difficulty falling/staying asleep | 4 |

**Total Score** = Sum of responses across all 25 items = **3 + 2 + 1 + ... + 4 = 62**

In this case, the patient’s SSS-CM total score is 62 out of 100, indicating moderate to high symptoms of severity.
