# Supplementary material for: Development and psychometric testing of symptom severity scale in older patients with cardiometabolic multimorbidity
Source: BMC Geriatr. 2025 Oct 14;25:771. doi: 10.1186/s12877-025-06370-1 (PMC12522849; doi:10.1186/s12877-025-06370-1)
Supplement: Supplementary file 3 — Supplementary Material 3 [file 12877_2025_6370_MOESM3_ESM.docx]

# Supplementary Material 3. Comparison of the SSS-CM with Existing Symptom Assessment Instruments

This table provides a comparative overview of the Symptom Severity Scale in Cardiometabolic Multimorbidity (SSS-CM) in relation to commonly used generic and disease-specific symptom assessment tools. It highlights the differences in scope, target population, and clinical practicality, particularly in the context of older adults with cardiometabolic multimorbidity.

| **Tool** | **Target Population** | **Scope** | **Disease-Specific** | **Covers Cardiovascular, Neurological, and Metabolic Symptoms** | **Designed for Older Adults** |
| --- | --- | --- | --- | --- | --- |
| Edmonton Symptom Assessment Scale (ESAS) | Palliative/Oncology | General symptom burden | No | No (limited to pain, appetite, fatigue) | No |
| Memorial Symptom Assessment Scale (MSAS) | Cancer patients | Physical and psychological symptoms | No | No (no disease-specific coverage) | No |
| MD Anderson Symptom Inventory (MDASI) | Cancer/advanced illness | Treatment-related symptoms | No | No (oncology-related focus) | No |
| T2Diabetes Symptom Checklist | Type 2 Diabetes | Endocrine/metabolic symptoms | Yes | No (diabetes-specific only) | No |
| Ischemic Stroke Questionnaire | Stroke patients | Stroke-related symptoms | Yes | No (neurological only) | No |
| Cardiac Symptom Survey | CABG or CAD patients | Cardiovascular symptoms | Yes | No (cardiac-specific only) | No |
| SSS-CM (present scale) | Older adults with cardiometabolic multimorbidity | Broad, multimorbidity-focused | No (multi-disease) | Yes (holistic symptom coverage across systems) | Yes |

CAD = coronary artery disease; CABG = Coronary artery bypass graft
